# High-Risk Human Papillomavirus Is Associated with HIV Acquisition among South African Female Sex Workers

**DOI:** 10.1155/2011/692012

**Published:** 2011-07-03

**Authors:** Bertran Auvert, Dianne Marais, Pascale Lissouba, Kevin Zarca, Gita Ramjee, Anna-Lise Williamson

**Affiliations:** ^1^CESP INSERM-UVSQ U1018, 94807 Villejuif, France; ^2^Hôpital Ambroise Paré, AP-HP, 92100 Boulogne-Billancourt, France; ^3^Université de Versailles—Saint-Quentin, 78035 Versailles, France; ^4^Faculty of Health Sciences, University of Cape Town, Cape Town 7701, South Africa; ^5^HIV Prevention Research Unit, MRC, Durban 3630, South Africa; ^6^IIDMM, University of Cape Town, Cape Town 7701, South Africa; ^7^NHLS, Groote Schuur Hospital, Cape Town 7937, South Africa

## Abstract

*Background*. Mounting evidence suggests an association between human papillomavirus (HPV) and HIV acquisition. This study aimed to explore this association among South African female sex workers (FSWs). *Methods*. We used data from 88 HIV-negative FSWs who participated in a vaginal gel (COL-1492) trial. Cervicovaginal rinse samples, obtained before HIV-seroconversion, were genotyped into high-risk (HR-) and low-risk (LR-) HPV. HIV-adjusted hazard ratios (aHRs) and 95% confidence intervals (CI) were estimated using Cox survival analysis. *Results*. HR- and LR-HPV prevalences were 70.5% (95% CI : 60.5–79.2) and 60.2% (95% CI : 49.9–70.0), respectively. Twenty-five women HIV seroconverted. Controlling for background characteristics and other sexually transmitted infections, HIV aHR increased by a factor of 1.7 (95% CI : 1.01–2.7, *P*_linear trend_ = 0.045) for an increase of one unit of the number of HR-HPV genotypes. *Conclusions*. HIV seroconversion among FSWs is associated with genital HR-HPV infection. Further investigation is warranted, including testing the possible protective effect of available HPV vaccines on HIV acquisition.

## 1. Background

HIV prevalence in South Africa is one of the highest in the world, with over 17% of adults infected with HIV [1]. Women are a particularly vulnerable group, with HIV prevalence and incidence far exceeding that of men from the region and of women in other parts of the world [2]. In South Africa, 90% of new infections in the 15 to 24 age group are among women [3], and HIV prevalences of up to 66.7% have been reported among women aged 24 [4]. 

A number of sexually transmitted infections (STIs), notably HSV-2, have been identified as key biomedical risk factors for the heterosexual transmission of HIV [2, 5, 6]. Among high-risk populations, female sex-workers (FSWs) generally have disproportionately high rates of STIs [7], and are likely to be at increased risk of HIV exposure. In low-income settings where condom use is inconsistent and access to effective STI treatment limited, an estimated 50 to 75% of FSWs are infected with at least one STI at any one time, and multiple infections are common [7]. 

In recent years, the association between HIV and genital human papillomavirus (HPV), one of the most common STI worldwide, has generated much interest. In South Africa, genital HPV is thought to affect about 21.0% of women with normal cytology [8], and HPV prevalence among FSWs is estimated to be two and a half to four times higher [9]. Genital HPV genotypes are divided into “high-risk” (HR) and “low-risk” (LR) types on the basis of their association with malignancies of the cervix. HR-HPV is a significant cause of morbidity and mortality in South Africa due to its aetiological association with genital cancer [10–15], cervical carcinoma being the second leading cause of cancer death among South African women [8]. 

Mounting evidence suggests an association between HPV serostatus and HIV infection. Several studies conducted among sub-Saharan African women indicate that HIV seropositivity is strongly associated with higher HPV prevalence [14, 16–23], higher HR-HPV prevalence [16, 17, 19], higher HPV incidence [20], a reduced likelihood of HPV infection clearance, which is associated with an increased risk of HPV-related lesions [20, 23, 24], and higher prevalence of infection with multiple HPV genotypes [14, 16, 17, 19, 21, 23, 24]. Furthermore, HIV seropositivity has also been found to be associated with the development of high-grade cervical squamous intraepithelial lesions [25], the presence of high and low-grade cervical squamous intraepithelial lesions [22], as well as invasive cervical carcinoma, a recognised AIDS-defining condition [19, 26] and the most common cancer among HIV-positive women in sub-Saharan Africa [27]. 

More recently, an association between HPV infection and HIV acquisition has been suggested, but to date, only few studies have been conducted among women to test this hypothesis [28–30]. The objective of this study was to assess HPV as a risk factor of HIV acquisition among FSWs in South Africa, a group which is presumably at high risk for STIs such as HPV and HIV.

## 2. Methods

### 2.1. Study Population and Sample Collection

Data were obtained from South African FSWs from the Kwazulu-Natal Midlands, who were participating in a multicentre, randomised, placebo-controlled, triple-blinded, phase 2/3 efficacy trial of COL-1492, a low-dose nonoxynol-9 microbicide. The trial was funded by the United Nations Programme on HIV/AIDS (UNAIDS), and conducted between September 1996 and June 2000. 

In brief, 187 healthy, nondrug using, not pregnant, HIV-negative South African FSWs were recruited by the Medical Research Council of Durban from truck stops along the major highway between the port city of Durban and the commercial capital Johannesburg and enrolled in the trial. At the screening visit, comprehensive information on the trial was provided and written informed consent was obtained. Participants were randomly assigned to a nonoxynol-9 containing gel (COL-1492) or the same placebo gel without nonoxynol-9. Data on demographics, background characteristics and sexual behaviour were collected at baseline. The primary endpoint was incident HIV-1 infection. The retention rate in the study at 48 weeks was 88%. Due to issues with processing, HPV sample data were unavailable for 83 women, but their baseline characteristics did not differ significantly from those of the remaining 104 women [31]. Details on the trial have been published elsewhere [31, 32]. 

Samples used for the present study were cervicovaginal rinses (CVRs), which were collected and tested for HPV. Theses samples were obtained at baseline, which started in 1996, in December 1998 (Followup A) and in December 1999 (Followup B). The full follow-up schedule of the study participants has been previously described [32, 33]. Full sets of HPV samples (i.e., one sample collected at each time point) were not obtained for all participants, some of whom having missing data. Infection statuses at baseline for *Trichomonas vaginalis*, *Candida albicans, Neisseria gonorrhoeae*, *Chlamydia trachomatis,* and syphilis were also obtained as described elsewhere [32]. 

In the current study, we included the 88 women who had at least one HPV result, obtained before HIV conversion for those who seroconverted.

### 2.2. Laboratory Testing

The detailed testing procedures have been previously described [31, 32]. 

HIV testing was performed with sensitive antibody ELISA (Abbott, Chicago, Ill, USA and Omnimed, UK) and confirmed by line immunoassay (Innogenetics, Ghent, Belgium). Seroconversion dates were established using p24 antigen detection ELISA and a DNA PCR assay. 

HPV DNA was extracted from the collected cervical cells using a Qiagen DNA mini kit (Qiagen, Calif, USA). HPV typing was performed with a reverse line blot assay (Roche Molecular Systems, Calif), according to a published method [34]. This assay could detect 13 HR-HPV genotypes (16, 18, 31, 33, 35, 39, 45, 51, 52, 56, 58, 59, and 68), 14 LR-HPV genotypes (6, 11, 26, 40, 42, 53, 54, 55, 57, 66, 73, 82, 83, and 84) and had probes for internal *β* globin control. As recommended by the manufacturer, *β* globin negative samples were not included in the analysis [35]. Samples were tested blinded of other study data. For quality control procedures, PCR controls for the HPV genotyping test were run together with test specimens in both the amplification and detection step of the assay.

Laboratory testing procedures for other STIs have been previously described [32] and are available at http://image.thelancet.com/extra/02art3365webmethods.pdf. 

### 2.3. Statistical Analyses

HIV-adjusted hazard ratios (aHRs) and 95% confidence intervals (CIs) were estimated with survival analysis (Cox model), using as covariates the following baseline characteristics: number of HR-HPV genotypes (referred to as number of HR-HPV in the text), number of LR-HPV genotypes (referred to as number of LR-HPV in the text), age (less than 24 years, 24 years, or more), intervention group (placebo or Nonoxynol-9), condom use (never, sometimes, or often), anal sex (yes or no), length of sex work (less than 24 months, 24 or more), number of clients per week (less than 20, 20 or more) and status of other STI (*Trichomonas vaginalis*, *Candida albicans, Neisseria gonorrhoeae*, *Chlamydia trachomatis,* and syphilis). The variables age, length of sex work, and number of clients were dichotomized based on their respective median values.

Because infections with HR-HPV and LR-HPV were correlated (*P* = 0.007, Spearman's rho test), the effect of LR-HPV on HIV incidence was first tested by controlling for HR-HPV. Similarly, the effect of HR-HPV on HIV incidence was first tested by controlling for LR-HPV.

The CIs of the percentages were calculated using Bayesian estimation [36]. Continuous variables and dichotomous variables were compared using the Kruskal-Wallis test and Fisher's exact test, respectively. Statistical analyses were performed using the statistical package SPSS for Windows version 8 (SPSS, Chicago, Ill, USA).

## 3. Results

### 3.1. Background Characteristics

Median age of the participants was 24 (min–max: 19–45; interquartile range (IQR): 19 to 30). Participants had been engaging in sex work for a median duration of 2.0 years (min–max: 0.17–23; IQR: 1.6 to 3.0), and their median number of clients per week was 20 (min–max: 5–35; IQR: 11 to 25). Median duration of participation in the study was 2.5 years (IQR: 1.7 to 2.8). Other background characteristics and STI status are indicated in Table 1.

### 3.2. HPV Infection and Genotype Distribution

For the analyses, we used the first available HPV testing, which was obtained at screening for 33 women (37.5%; 33/88), in December 1998 for 44 women (50.0%, 44/88) for whom HPV data at screening were unavailable, and in December 1999 for 11 women (12.5%; 11/88) for whom HPV data were unavailable at screening and in December 1998. The HR- and LR-HPV prevalences were 70.5% (62/88, 95% CI: 60.5–79.2) and 60.2% (53/88, 95% CI: 49.9–70.0), respectively. 

**Figure 1:**
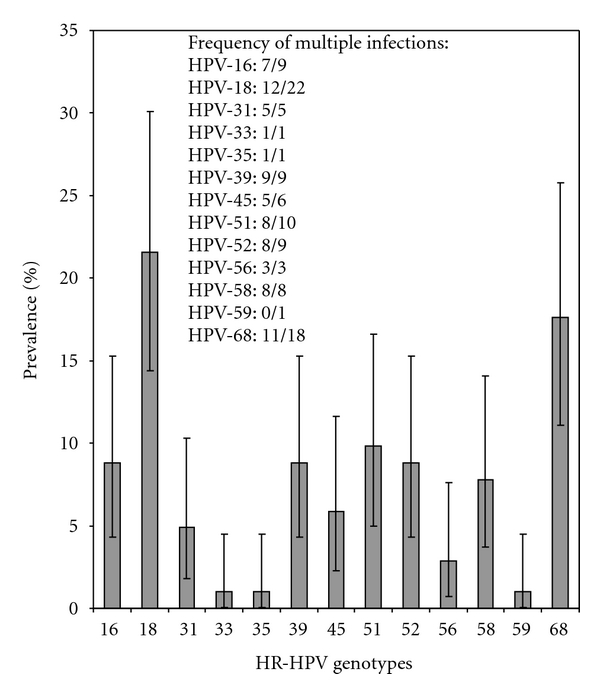
Distribution of the 102 high-risk human papillomavirus (HR-HPV) genotypes identified in the sample and frequency of multiple infections per HR-HPV genotype.

The distribution of the numbers of HR-HPV and LR-HPV is shown in Table 2. Among the 62 women infected with at least one HR-HPV, 102 different HR-HPV genotypes were detected. The distribution of these genotypes is illustrated in Figure 1. This figure shows that among the 13 high-risk genotypes detected, HR-HPV 18 was the most common. HR-HPV 16 and HR-HPV 18 together represented close to one third of all HR-HPV genotypes detected among the participants.

### 3.3. HIV Acquisition and Risk Factors

Among the 88 women, 28.4% (25/88) HIV seroconverted during followup. When controlling for the number of HR-HPV, the number of LR-HPV was not a risk factor for HIV acquisition (aHR = 0.95, 95% CI: 0.68–1.3, *P*_linear  trend_ = 0.76). In contrast, when controlling for the number of LR-HPV, the number of HR-HPV was a risk factor for HIV acquisition (aHR = 1.6, 95% CI: 1.1–2.2, *P*_linear  trend_ = 0.019). When assessing HIV acquisition as a function of the total number of genotypes, regardless of type, the univariate effect was HR=1.2 (95% CI: 0.97–1.4; *P* = 0.089), and the multivariate effect was aHR = 1.1 (95% CI: 0.84–1.4; *P* = 0.51).

The mean (median) duration in years of followup before HIV infection was 2.4 (2.7) for women not infected with HR-HPV and 2.2 (2.5) for those infected with at least one HR-HPV genotype. The effect of HR-HPV status and other covariates on HIV acquisition, and the distribution of HIV cases, are indicated in Table 1. In the multivariate analysis, HIV acquisition significantly increased with the number of HR-HPV genotypes and level of education, and significantly decreased with age. Women infected with two or more HR-HPV genotypes were at higher risk of acquiring HIV than those infected with zero or one HR-HPV genotype (aHR = 4.0, 95% CI: 1.2–14.0, *P* = 0.028). 

**Figure 2:**
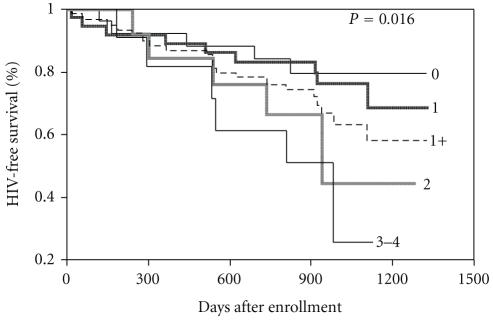
Kaplan-Meier HIV-free survival estimates for women with 0, 1, 2, 3-4, and at least 1 (1+) high-risk human papillomavirus genotypes.

As shown in Figure 2, HIV-free survival decreased with the number of HR-HPV genotypes.

A sensitivity analysis was conducted to examine the effect of HR-HPV on HIV acquisition among women in the placebo group. The univariate effect was HR=1.9 (95% CI: 1.1–3.3, *P* = 0.027). The corresponding multivariate analysis only included covariates having a univariate *P* value lower than 0.15 (age and education) because of the smaller number of HIV infections and yielded an effect aHR = 1.8 (95% CI: 1.0–3.3, *P* = 0.041).

### 3.4. Estimation of Potential Biases

Using the first available HPV testing result for our analysis may have been a source of bias because in doing so, it was assumed that HPV status at baseline and at the two follow-up visits were equivalent. This is not necessarily correct, because the observed association between HPV and HIV acquisition could be time dependent due to the transient nature of HPV infection. To address this issue, it was recommended that the analyses be repeated taking time zero as the date of the first HPV test. Although this approach reduced the power of the analysis because it reduced the total length of followup, the multivariate effect obtained (aHR = 1.6; 95% CI: 0.97 to 2.6; *P* = 0.067) was very close to the reported estimate from the main analysis.

The 16 (104 minus 88) women who were excluded due to lack of HPV results were compared with the 88 remaining women in terms of background characteristics and STI status, which are listed, Table 1. They did not differ with respect to their average age (26.6 versus 25.8; *P* = 0.54), percentage having been randomized to the intervention group (50.0% versus 46.6%; *P* = 1.0), percentage with at least six years of education (81.3% versus 59.1%; *P* = 0.16), percentage using condoms often (31.3% versus 19.3%; *P* = 0.32), percentage practicing anal sex (43.8% versus 43.2%; *P* = 1.0), average duration of sex work in months (27.0 versus 34.9; *P* = 0.59), average number of clients per week (21.4 versus 18.7; *P* = 0.26), prevalence of *Trichomonas vaginalis* (18.8% versus 35.8%; *P* = 0.25), prevalence of *Candida albicans* (50.0% versus 37.0%; *P* = 0.40), prevalence of *Neisseria gonorrhoeae* (12.5% versus 8.8%; *P* = 0.64), and prevalence of Syphilis (25.0% versus 26.8%; *P* = 1.0). However, the difference in the prevalence of *Chlamydia trachomatis* was borderline (35.7% versus 13.5%; *P* = 0.058). The statistical power for these analyses may have been limited due to the small samples sizes.

## 4. Discussion

While the burden of disease associated with HIV and HPV infection is significant, especially in sub-Saharan Africa, there is limited research on the link between HPV and HIV acquisition. In the present study, we found that HR-HPV was significantly and independently associated with HIV acquisition among South African FSWs and that the risk of HIV infection significantly increased in the event of multiple HR-HPV infections. No association was found with LR-HPV.

To our knowledge, only six published observational studies, three conducted among men and three among women, have explored HPV infection and HIV acquisition [28–30, 37–39]. All but one study [38] were conducted in sub-Saharan Africa, where HIV transmission is predominantly heterosexual. In five of these studies, HIV seroconversion was associated with HR-HPV. Only two studies found an association between LR-HPV and HIV acquisition. 

One study [38], in which did HPV was not assessed by type, reported that anal HPV infection was associated with HIV acquisition among men who have sex with men (aHR = 3.5, 95% CI: 1.2–10.6). Another study [37] found that HIV acquisition was facilitated among HR-HPV infected South African men (adjusted incidence rate ratio= 3.8, 95% CI: 1.8–7.7), but not among those infected with LR-HPV. A third study conducted among Kenyan men [39] reported an independent, increased risk of HIV seroconversion among HPV-positive men (aHR = 1.8, 95% CI: 1.1–2.9) but not when HR and LR-HPV were assessed separately. Among women, a case-control study [30] reported that for Zimbabwean women with cervical HPV infection, the odds of acquiring HIV were over twice higher than among HR-HPV and LR-HPV uninfected women after adjustment for behavioural and biological factors (aOR = 2.3, 95% CI: 1.4–3.9 and aOR = 2.8, 95% CI: 1.3–5.9, resp.). Another study conducted in Zimbabwe [28] indicated that concurrent genital HR-HPV (assessed at the time of HIV detection) and recent HR-HPV infection (assessed within six months of HIV detection) approximately doubled the risk of HIV acquisition independently of sexual risk factors and other STIs (aHR = 2.0, 95% CI: 1.2–3.3 and aHR = 2.0, 95% CI: 1.2–3.2, resp.). This study also found a significant association between concurrent LR-HPV infection and HIV acquisition (aHR = 1.7, 95% CI: 1.0–2.9). Lastly, the cohort study conducted among Rwandan FSWs [29] found that women who HIV seroconverted were about five times more likely to have been infected with HR-HPV at baseline (OR = 4.9, 95% CI: 1.2–19.7). 

Our study findings are mostly coherent with these reports; however, comparisons are difficult because of substantial variations in terms of sample population, covariate adjustment and HPV status assessment. For instance, only one study assessed the association among individuals with risky sexual behaviour [29] but reported unadjusted estimates. The other five studies [28, 30, 37–39] controlled for HSV-2 infection but only three [28, 30, 37] for condom use and another three for other STIs status [28, 30, 38]. The most notable variations concerned the timing of HPV-infection status assessment, which varied from baseline [28, 38, 39], to six months after recruitment [29], within three [30] or six months [28] before HIV seroconversion, concurrently with HIV detection [28] or after HIV seroconversion [37]. Due the transient nature of HPV infections, it is plausible that the association between HPV and HIV acquisition is time dependent, and it could explain the variations in study findings. Differing study designs may also explain the fact that we did not detect an increased risk of HIV acquisition with LR-HPV infection, as it had been reported by two of the studies [28, 30], but it could also reflect specific HPV-type differences in host response.

Several nonexclusive and plausible explanations can account for our findings. Firstly, HIV and HPV are sexually transmitted viruses which share the same transmission route and behavioral risk factors. However, the association observed remains largely significant even after controlling for sexual behavioral covariates although residual confounding cannot be excluded. Secondly, it could be argued that HIV-HPV coinfected male partners may have shed HR-HPV more intensively than HIV-negative male partners. Indeed, in the case of a depressed immune system, HPV lesions are more likely to be dysplastic [40], and immunodepression can reactivate latent HPV infections [41–43]. Since HPV is assumed to be more infectious than HIV, it is possible that women exposed to both infections at the same time acquire HPV before HIV, and that the hypothesized effect of HPV infection on HIV acquisition be due to residual confounding. This would be an issue even if the ascertainment of HPV among exposed partners occurred before HIV seroconversion. Thirdly, the findings could simply indicate that HR-HPV facilitates HIV acquisition, which seems the most likely explanation, since no effect of LR-HPV was detected. Lastly, as in any statistical analysis, our results could be due to chance. However, the numbers of HR and LR infections are similar. Thus, the powers of the individual analyses of the effect of HR- and LR-HPV on HIV acquisition are also similar. The probability that our results are due to chance cannot be excluded, but we think that it is reasonably small.

Several factors could account for a causal association of HR-HPV with HIV acquisition. first, their association is very strong, as shown in observational studies. Secondly, causality is biologically plausible: HPV could disrupt the local and systemic immune systems and predispose to HIV infection [27], either via the recruitment of HIV target cells [44, 45], which would facilitates its acquisition, or cytokine stimulation [45, 46], which could increase HIV replication. Thus, since HR-HPV and LR-HPV are believed to elicit a differential immune response [47], it would explain the lack of association between LR-HPV and HIV as found in this study and in a cross-sectional survey conducted in Zambia [16]. Thirdly, HPV could lead to genital lesions that may facilitate HIV acquisition. Unfortunately, we did not have the necessary data on HPV-associated lesions to assess this hypothesis. Further research is needed to clarify the etiological mechanisms of the association between HPV and HIV incidence. 

This study has several limitations: First, its observational nature does not allow the interpretation of the findings as a causal association between HR-HPV status and HIV acquisition. Secondly, we did not obtain sets of samples collected at the same time for all participants although only a small proportion were not included because of missing data. Thirdly, HSV-2 status, anal HPV sampling, and cervical cytology were not available, and thus their effects on the observed association could not be tested. In particular, HSV-2 has been hypothesized to facilitate HIV acquisition [48], and could potentially play a mediating role. However, among the six identified studies exploring the link between HPV and HIV seroconversion, five [28, 30, 37–39] found a significant association even when HSV-2 status was included as a covariate. Fourthly, HPV infections are known to be transient [49], and this may have diluted the associations observed in this study. Lastly, the sample of women used in this study was not a representative sample. Nevertheless, there are few reasons to think that these limitations could invalidate the observed association between HR-HPV status and HIV acquisition.

## 5. Conclusion

Despite these limitations, these findings indicate that the hypothesis of a facilitating effect of HR-HPV on HIV acquisition requires further investigation using longitudinal data. The validation of this hypothesis could necessitate testing the potential protective effect of available HPV vaccines on HIV acquisition.

## Figures and Tables

**Table 1:** Distribution of detected HIV cases and risk factors of HIV acquisition.

Factor	Distribution	Number of HIV infections	Mean survival (days)	Univariate HR (95% CI)	Multivariate aHR* (95% CI)
Number of high-risk HPV					
0	29.5% (26/88)	5	1148		
1	43.2% (38/88)	9	1110	1.5 (1.1 to 2.1) *P* = 0.016**	1.7 (1.01 to 2.7) *P* = 0.045**
2	14.8% (13/88)	5	934		
3-4	12.5% (11/88)	6	775		
Number of low-risk HPV					
0	39.8% (35/88)	7	1134		
1	35.2% (31/88)	11	992	1.1 (0.12 to 0.69) *P* = 0.70**	0.77 (0.49 to 1.2) *P* = 0.26**
2	15.9% (14/88)	5	932		
3	9.1 (8/88)	2	1136		
Age (years)					
Less than 24	46.6% (41/88)	18	933	1	1
24 or more	53.4% (47/88)	7	1192	0.29 (0.12 to 0.69) *P* = 0.005	0.28 (0.079 to 0.97) *P* = 0.044
Intervention					
Placebo	46.6% (41/88)	9	1132	1	1
Nonoxynol-9	53.4% (47/88)	16	1013	1.8 (0.81 to 4.2) *P* = 0.15	2.0 (0.75 to 5.1) *P* = 0.17
Education					
Less than 6 years	40.9% (36/88)	6	1184	1	1
6 years or more	59.1% (52/88)	19	990	2.7 (1.1 to 6.8) *P* = 0.033	5.5 (1.6 to 19.6) *P* = 0.0082
Condom use					
Never or sometimes	80.7% (71/88)	22	1044	1	1
Often	19.3% (17/88)	3	1175	0.57 (0.17 to 1.9) *P* = 0.36	0.29 (0.05 to 1.6) *P* = 0.16
Anal sex					
No	56.8% (50/88)	18	1029	1	1
Yes	43.2% (38/88)	7	1137	0.53 (0.22 to 1.3) *P* = 0.16	0.35 (0.12 to 1.0) *P* = 0.052
Length of sex work (months)					
Less than 24	50.0% (44/88)	13	1045	1	1
24 or more	50.0% (44/88)	12	1091	0.89 (0.41 to 1.9) *P* = 0.77	1.5 (0.41 to 5.6) *P* = 0.53
Number of clients per week					
Less than 20	46.6% (41/88)	13	1056	1	1
20 or more	53.4% (47/88)	12	1076	0.78 (0.36 to 1.7) *P* = 0.54	1.4 (0.48 to 3.9) *P* = 0.56
*Trichomonas vaginalis*					
Negative	64.2% (52/81)	17	1030	1	1
Positive	35.8% (29/81)	6	1109	0.52 (0.21 to 1.3) *P* = 0.17	1.4 (0.41 to 4.6) *P* = 0.61
*Candida albicans*					
Negative	63.0% (51/81)	16	1038	1	1
Positive	37.0% (30/81)	7	1129	0.67 (0.28 to 1.6) *P* = 0.38	0.55 (0.18 to 1.7) *P* = 0.29
*Neisseria gonorrhoeae*					
Negative	91.3% (73/80)	20	1074	1	1
Positive	8.8% (7/80)	3	1065	1.33 (0.40 to 4.5) *P* = 0.54	2.3 (0.53 to 9.6) *P* = 0.27
*Chlamydiatrachomatis*					
Negative	86.5% (64/74)	20	1039	1	1
Positive	13.5% (10/74)	7	1120	0.77 (0.23 to 2.6) *P* = 0.67	0.25 (0.06 to 1.1) *P* = 0.070

Syphilis					
Negative	73.2% (60/82)	20	1042	1	1
Positive	26.8% (22/82)	4	1138	0.50 (0.17 to 1.5) *P* = 0.21	0.66 (0.17 to 2.6) *P* = 0.55

(a) HR: (adjusted) hazard ratio.

HPV: human papillomavirus.

*The following variables were included in the multivariate analysis: number of high-risk HPV, number of low-risk HPV, age, intervention group, education, condom use, anal sex, length of sex work, number of clients per week, infection with Trichomonas vaginalis, infection with Candida albicans, infection with Neisseria gonorrhoea, infection with Chlamydia trachomatis, and infection with syphilis.

**Increase of hazard ratio of HIV acquisition for an increase of one unit of the number of HPV. The *P* value corresponds to a linear trend.

**Table 2:** Distribution of the number of human papillomavirus (HPV) genotypes among HIV-negative women.

Number of high-risk HPV genotypes	Percentage of women (95% CI) [*n*]	Number of low-risk HPV genotypes	Percentage of women (95% CI) [*n*]
0	29.5 (20.8–39.5) [26]	0	39.8 (30.0–50.1) [35]
1	43.2 (33.2–53.5) [38]	1	35.2 (25.8–45.4) [31]
2	14.8 (8.4–23.1) [13]	2	15.9 (9.3–24.4) [14]
3	10.2 (5.1–17.7) [9]	3	3.4 (0.84–8.7) [3]
4	2.3 (0.36–7.0) [2]	4	5.7 (2.1–11.9) [5]
1–4	70.5 (60.5–79.2) [62]	1–4	60.2 (49.9–70.0) [53]

Total	100 [88]	Total	100 [88]

## References

[1] UNAIDS Global Report: UNAIDS Report on the global AIDS epidemic 2010. http://www.unaids.org/globalreport/Global_report.htm.

[2] Chersich MF, Rees HV (2008). Vulnerability of women in southern Africa to infection with HIV: biological determinants and priority health sector interventions. *AIDS*.

[3] Rehle TM, Shisana O, Pillay V, Zuma K, Puren A, Parker W (2007). National HIV incidence measures—new insights into the South African epidemic. *South African Medical Journal*.

[4] Auvert B, Ballard R, Campbell C (2001). HIV infection among youth in a South African mining town is associated with herpes simplex virus-2 seropositivity and sexual behaviour. *AIDS*.

[5] Joelle ST, Taljaard D, Lissouba P (2009). Effect of HSV-2 serostatus on acquisition of HIV by young men: results of a longitudinal study in orange farm, South Africa. *Journal of Infectious Diseases*.

[6] White RG, Orroth KK, Glynn JR (2008). Treating curable sexually transmitted infections to prevent HIV in Africa: still an effective control strategy?. *Journal of Acquired Immune Deficiency Syndromes*.

[7] Steen R, Dallabetta G (2003). Sexually transmitted infection control with sex workers: regular screening and presumptive treatment augment efforts to reduce risk and vulnerability. *Reproductive Health Matters*.

[8] WHO-ICO (2010). *Human Papillomavirus and Related Cancers: Summary Report Update*.

[9] Smith JS, Melendy A, Rana RK, Pimenta JM (2008). Age-specific prevalence of infection with human papillomavirus in females: a global review. *Journal of Adolescent Health*.

[10] Castellsague X (2008). Natural history and epidemiology of HPV infection and cervical cancer. *Gynecologic Oncology*.

[11] Bosch FX, Manos MM, Munoz N (1995). Prevalence of human papillomavirus in cervical cancer: a worldwide perspective. International biological study on cervical cancer (IBSCC) Study Group. *Journal of the National Cancer Institute*.

[12] Schiffman M, Castle PE, Jeronimo J, Rodriguez AC, Wacholder S (2007). Human papillomavirus and cervical cancer. *Lancet*.

[13] Castellsague SDSX, Aguado T, Louie KS (2007). HPV and cervical cancer in the world. *Vaccine*.

[14] Banura C, Franceschi S, Van Doorn LJ (2008). Infection with human papillomavirus and HIV among young women in Kampala, Uganda. *Journal of Infectious Diseases*.

[15] Walboomers JMM, Jacobs MV, Manos MM (1999). Human papillomavirus is a necessary cause of invasive cervical cancer worldwide. *Journal of Pathology*.

[16] Ng’andwe C, Lowe JJ, Richards PJ, Hause L, Wood C, Angeletti PC (2007). The distribution of sexually-transmitted Human Papillomaviruses in HIV positive and negative patients in Zambia, Africa. *BMC Infectious Diseases*.

[17] Safaeian M, Kiddugavu M, Gravitt PE (2008). Prevalence and risk factors for carcinogenic human papillomavirus infections in rural Rakai, Uganda. *Sexually Transmitted Infections*.

[18] Baay MFD, Kjetland EF, Ndhlovu PD (2004). Human papillomavirus in a rural community in Zimbabwe: the impact of HIV co-infection on HPV genotype distribution. *Journal of Medical Virology*.

[19] Didelot-Rousseau MN, Nagot N, Costes-Martineau V (2006). Human papillomavirus genotype distribution and cervical squamous intraepithelial lesions among high-risk women with and without HIV-1 infection in Burkina Faso. *British Journal of Cancer*.

[20] Safaeian M, Kiddugavu M, Gravitt PE (2008). Determinants of incidence and clearance of high-risk human papillomavirus infections in rural Rakai, Uganda. *Cancer Epidemiology Biomarkers and Prevention*.

[21] Marais DJ, Passmore JAS, Denny L, Sampson C, Allan BR, Williamson AL (2008). Cervical and oral human papillomavirus types in HIV-1 positive and negative women with cervical disease in South Africa. *Journal of Medical Virology*.

[22] Yamada R, Sasagawa T, Kirumbi LW (2008). Human papillomavirus infection and cervical abnormalities in Nairobi, Kenya, an area with a high prevalence of human immunodeficiency virus infection. *Journal of Medical Virology*.

[23] Banura C, Franceschi S, Van Doorn LJ (2008). Prevalence, incidence and clearance of human papillomavirus infection among young primiparous pregnant women in Kampala, Uganda. *International Journal of Cancer*.

[24] Rowhani-Rahbar A, Hawes SE, Sow PS (2007). The impact of HIV status and type on the clearance of human papillomavirus infection among Senegalese women. *Journal of Infectious Diseases*.

[25] Hawes SE, Critchlow CW, Sow PS (2006). Incident high-grade squamous intraepithelial lesions in senegalese women with and without human immunodeficiency virus type 1 (HIV-1) and HIV-2. *Journal of the National Cancer Institute*.

[26] Kahesa C, Mwaiselage J, Wabinga HR, Ngoma T, Kalyango JN, Karamagi CAS (2008). Association between invasive cancer of the cervix and HIV-1 infection in Tanzania: the need for dual screening. *BMC Public Health*.

[27] Clarke B, Chetty R (2002). Postmodern cancer: the role of human immunodeficiency virus in uterine cervical cancer. *Journal of Clinical Pathology*.

[28] Smith-McCune KK, Shiboski S, Chirenje MZ (2010). Type-specific cervico-vaginal human papillomavirus infection increases risk of HIV acquisition independent of other sexually transmitted infections. *PLoS ONE*.

[29] Veldhuijzen NJ, Vyankandondera J, Van De Wijgert JH (2010). HIV acquisition is associated with prior high-risk human papillomavirus infection among high-risk women in Rwanda. *AIDS*.

[30] Averbach SH, Gravitt PE, Nowak RG (2010). The association between cervical human papillomavirus infection and HIV acquisition among women in Zimbabwe. *AIDS*.

[31] Marais DJ, Carrara H, Ramjee G, Kay P, Williamson AL (2009). HIV-1 seroconversion promotes rapid changes in cervical human papillomavirus (HPV) prevalence and HPV-16 antibodies in female sex workers. *Journal of Medical Virology*.

[32] Van Damme L, Ramjee G, Alary M (2002). Effectiveness of COL-1492, a nonoxynol-9 vaginal gel, on HIV-1 transmission in female sex workers: a randomised controlled trial. *Lancet*.

[33] Marais D, Carrara H, Kay P, Ramjee G, Allan B, Williamson AL (2006). The impact of the use of COL-1492, a nonoxynol-9 vaginal gel, on the presence of cervical human papillomavirus in female sex workers. *Virus Research*.

[34] Gravitt PE, Peyton CL, Alessi TQ (2000). Improved amplification of genital human papillomaviruses. *Journal of Clinical Microbiology*.

[35] Gravitt PE, Peyton CL, Apple RJ, Wheeler CM (1998). Genotyping of 27 human papillomavirus types by using L1 consensus PCR products by a single-hybridization, reverse line blot detection method. *Journal of Clinical Microbiology*.

[36] Newcombe RG (1998). Two-sided confidence intervals for the single proportion: comparison of seven methods. *Statistics in Medicine*.

[37] Auvert B, Lissouba P, Cutler E, Zarca K, Puren A, Taljaard D (2010). Association of oncogenic and nononcogenic human papillomavirus with HIV incidence. *Journal of Acquired Immune Deficiency Syndromes*.

[38] Chin-Hong PV, Husnik M, Cranston RD (2009). Anal human papillomavirus infection is associated with HIV acquisition in men who have sex with men. *AIDS*.

[39] Smith JS, Moses S, Hudgens MG (2010). Increased risk of HIV acquisition among kenyan men with human papillomavirus infection. *Journal of Infectious Diseases*.

[40] Steben M, Duarte-Franco E (2007). Human papillomavirus infection: epidemiology and pathophysiology. *Gynecologic Oncology*.

[41] Aubin F, Pretet JL, Mougin C, Riethmuller D (2007). Human papillomavirus infection. *Annales de Dermatologie et de Venereologie*.

[42] Aynaud O, Piron D, Barrasso R, Poveda JD (1998). Comparison of clinical, histological, and virological symptoms of HPV in HIV-1 infected men and immunocompetent subjects. *Sexually Transmitted Infections*.

[43] Strickler HD, Burk RD, Fazzari M (2005). Natural history and possible reactivation of human papillomavirus in human immunodeficiency virus-positive women. *Journal of the National Cancer Institute*.

[44] Coleman N, Birley HDL, Renton AM (1994). Immunological events in regressing genital warts. *American Journal of Clinical Pathology*.

[45] Nicol AF, Gomes Fernandes AT, Grinsztejn B (2005). Distribution of immune cell subsets and cytokine-producing cells in the uterine cervix of human papillomavirus (HPV)-infectecl women: influence of HIV-1 coinfection. *Diagnostic Molecular Pathology*.

[46] Gage JR, Sandhu AK, Nihira M (2000). Effects of human papillomavirus-associated cells on human immunodeficiency virus gene expression. *Obstetrics and Gynecology*.

[47] Monk BJ, Tewari KS (2007). The spectrum and clinical sequelae of human papillomavirus infection. *Gynecologic Oncology*.

[48] Van de Perre P, Segondy M, Foulongne V (2008). Herpes simplex virus and HIV-1: deciphering viral synergy. *The Lancet Infectious Diseases*.

[49] Cuzick J, Arbyn M, Sankaranarayanan R (2008). Overview of human papillomavirus-based and other novel options for cervical cancer screening in developed and developing countries. *Vaccine*.

